# Grants to Hospitals Recommended by the Distribution Committee

**Published:** 1916-12-23

**Authors:** 


### Grants to Hospitals Recommended by the Distribution Committee.

*The Distribution Committee desire to draw attention to the fact that it must not be assumed that the absence of a grant implies dissatisfaction.*

ALEXANDRA Hospital for Children, £200.

BECKENHAM Cottage Hospital, £25. Belgrave Hospital, £1,500, of which £500 to extinguish building fund debt. Blackheath and Charlton Hospital, £100. Bolingbroke Hospital, £750; regret to see that the cost has increased in greater proportion than at similar hospitals. British Hospital for Mothers and Babies, Woolwich, £200; a grant of £1,500 was set aside in 1913 towards the building of the amalgamated hospital, in accordance with scheme to be approved.

CANNING TOWN Women's Settlement Hospital (late Medical Mission), £750, of which £250 to reduce debt. Central London Ophthalmic Hospital, £450. Central London Throat and Ear Hospital, capital grants amounting to £10,500 are set aside and promised for the purpose of an amalgamation of the Throat, Nose, and Ear Hospitals (see paragraph 8 of Report). Charing Cross Hospital, £6,000, of which £1,000 in reduction of mortgage debt and £1,500 to improvements in accordance with the scheme submitted to the Fund. Glad to note success of efforts to improve the financial position of the hospital, particularly in regard to annual subscriptions. Chelsea Hospital for Women, £2,500, of which £2,000 to reduce debt on rebuilding on new site in accordance with the scheme submitted to the Fund. Cheyne Hospital for Sick and Incurable Children, £200. City of London Hospital for Diseases of the Chest, Victoria Park, £4,500, of which £1,500 to reduce deficit on recent improvements in accordance with the scheme submitted to the Fund. City of London Lying-in Hospital, £600. Clapham Maternity Hospital, £1,000, of which £400 to extinguish mortgage debt.

DREADNOUGHT Hospital, £3,750.

EAST END Mothers' Lying-in Home, £400. East London Hospital for Children, £4,000, of which £1,500 to reduce debt (see paragraph 5 of Report). Eltham and Mottingham Cottage Hospital, £75.

FINCHLEY Cottage Hospital, £50. Florence Nightingale Hospital for Gentlewomen, £200. French Hospital, £200.

GENERAL Lying-in Hospital, £300. German Hospital, £100. Gordon Hospital for Fistula, etc. —. Great Northern Central Hospital, £5,000, of which £1,000 to reduce debt (see paragraph 5 of Report). Grosvenor Hospital for Women, £350. Guy's Hospital, £9,000, of which £2,000 to reduce deficit on recent building improvements in accordance with the scheme submitted to the Fund.

HAMPSTEAD General Hospital, £3,500, of which £1,600 to reduce debt, and £150 as agreed upon amalgamation with North-West London Hospital. Hendon Cottage Hospital, £25. Hornsey Cottage Hospital, £25. Hospital and Home for Incurable Children, Hampstead, £50, in consideration of the fact that curable cases are admitted. Hospital and Home for Sick Children, Sydenham, £300. Hospital for Consumption, Brompton (including Sanatorium at Frimley), £4,000. Hospital for Diseases of the Throat, £1,400, of which £900 is maintenance grant, as agreed, upon amalgamation with the London Throat Hospital, and £500 is a grant towards loss incurred by postponement of building scheme through the war. Hospital for Epilepsy and Paralysis, £1,250. Hospital for Sick Children, £4,000, of which £500 to reduce debt. Hos-

### KING EDWARD'S HOSPITAL FUND—

### Grants to Hospitals—(*continued*).

pital for Women, Soho Square, £1,750, of which £500 to reduce debt. Hospital of St. John and St. Elizabeth, £500.

INFANTS' Hospital, £200. Italian Hospital, £400.

KENSINGTON and Fulham General Hospital, £50. Kensington Dispensary and Children's Hospital, £25. King Edward Memorial Hospital (Ealing), £650. King's College Hospital, £5,500, of which £3,000 to reduction of debt on removal to South London, making £62,600 in all contributed by the Fund to this purpose.

LEYTON, Walthamstow, and Wanstead Children's and General Hospital, £500. London Fever Hospital, £200. London Homœopathic Hospital, £1,250, of which £250 to reduce general fund debt. London Hospital, £15,000, of which £2,000 to new nurses' home in accordance with the scheme submitted to the Fund. London Lock Hospital, £4,000, of which £2,000 to maintenance of Harrow Road Hospital, and £2,000 to sanitary improvements at Harrow Road in accordance with the scheme submitted to the Fund (see also paragraph 9 of Report). London Temperance Hospital, £2,000, of which £250 to reduction of debt on maintenance account.

MATERNITY Charity and District Nurses' Home, Plaistow, £300. Memorial Hospital, Mildmay Park, £200. Metropolitan Hospital, £4,000, of which £1,000 to reduce debt (see paragraph 5 of Report). Middlesex Hospital, £5,500, of which £1,000 to recent improvements. Middlesex Hospital Cancer Charity, £150. Mildmay Mission Hospital, £800, of which £300 to reduce debt. Miller General Hospital for South-East London, £1,600.

NATIONAL Hospital for Diseases of the Heart, £150. National Hospital for the Paralysed and Epileptic, £3,000, of which £500 to reduce debt. Nelson Hospital (late South Wimbledon, etc.), £400. New Hospital for Women, £800. Norwood Cottage Hospital, £100.

PADDINGTON Green Children's Hospital, £2,000, of which £200 to fire escape staircase and £1,000 to reduce debt. The grant in reduction of debt is on the understanding that a strenuous effort is made to secure adequate local support (see paragraph 5 of Report). Passmore Edwards Acton Cottage Hospital, £100. Passmore Edwards East Ham Hospital, £150, of which £75 to scheme submitted to the Fund. Passmore Edwards Hospital for Willesden, £50. Passmore Edwards Cottage Hospital, Wood Green, £50. Poplar Hospital, £200. Prince of Wales's General Hospital, £5,000, of which £2,000 to reduce general fund debt.

QUEEN CHARLOTTE'S Lying-in Hospital, £1,250, of which £250 to reduce debt. Queen Mary's Hospital for the East End, incorporating West Ham and Eastern General Hospital, £4,000 (see paragraph 5 of Report). Queen's Hospital for Children, £4,000, of which £1,250 to reduce debt.

ROYAL Dental Hospital of London, £400. Royal Eye Hospital, £350. Royal Free Hospital, £3,000. Royal Hospital for Diseases of the Chest, City Road, £1,250, to reduce debt. Royal Hospital, Richmond, £250. Royal London Ophthalmic Hospital, £2,500. Royal National Orthopædic Hospital, £1,250. Royal Waterloo Hospital for Children and Women, £2,000, of which £500 to reduce debt. Royal Westminster Ophthalmic Hospital, £50.

ST. ANDREW'S Hospital (Dollis Hill), £50. St. Columba's Hospital, £200, of which £100 to reduce debt. St. George's Hospital, £5,000. St. John's Hospital for Diseases of the Skin, —. St. John's Hospital, Lewisham, £300. St. Luke's House, £50. St. Mark's Hospital, £300. St. Mary's Hospital, £3,570. St. Mary's Hospital for Women and Children, Plaistow, £850. St. Monica's Home Hospital, £150, of which £50 towards erection of fire escape staircase. St. Peter's Hospital for Stone, £400, of which £150 to reduce debt on erection of fire escape staircases. St. Saviour's Hospital, Osnaburgh Street, £150. St. Thomas's Hospital. The application has been considered on the accounts for 1915, as the financial position in 1916 will not be known until next year (see paragraph 4 of Report). Salvation Army Maternity Hospital, £250. Samaritan Free Hospital, £2,000, of which £1,000 to reduce debt. Santa Claus Home, £50. Stoke Newington Home Hospital for Women, £100.

UNIVERSITY College Hospital, £5,000.

VICTORIA Hospital for Children, £1,000, of which £200 towards new heating apparatus.

WEST END Hospital for Nervous Diseases, £400. Western Ophthalmic Hospital, £600, of which £500 to reduction of debt on rebuilding of out-patient department. West London Hospital, £4,000, of which £500 to furnishing of new nurses' home. Westminster Hospital, £4,150, of which £3,850 to maintenance, and £300 annual grant to acquisition of new site as agreed. Wimbledon Hospital, £100. Woolwich and Plumstead Cottage Hospital, £25.

**Total Grants to Hospitals for the Year 1916, £162,500.**

